# Automated sensing and splitting of stem cell colonies on microraft arrays

**DOI:** 10.1063/1.5113719

**Published:** 2019-08-29

**Authors:** Matthew DiSalvo, Nicole M. Smiddy, Nancy L. Allbritton

**Affiliations:** 1 Joint Department of Biomedical Engineering, University of North Carolina at Chapel Hill and North Carolina State University, Chapel Hill/Raleigh, North Carolina 27599/27607, USA; 2 Department of Chemistry, University of North Carolina at Chapel Hill, Chapel Hill, North Carolina 27599, USA

## Abstract

Human induced pluripotent stem cells (hiPSCs) are widely used for disease modeling, tissue engineering, and clinical applications. Although the development of new disease-relevant or customized hiPSC lines is of high importance, current automated hiPSC isolation technologies rely largely on the fluorescent labeling of cells, thus limiting the cell line development from many applications. The objective of this research was to develop a platform for high-throughput hiPSC cytometry and splitting that utilized a label-free cell sensing approach. An image analysis pipeline utilizing background subtraction and standard deviation projections was implemented to detect hiPSC colonies from bright-field microscopy data. The pipeline was incorporated into an automated microscopy system coupling quad microraft cell-isolation arrays, computer-based vision, and algorithms for smart decision making and cell sorting. The pipeline exhibited a hiPSC detection specificity of 98% and a sensitivity of 88%, allowing for the successful tracking of growth for hundreds of microcolonies over 7 days. The automated platform split 170 mother colonies from a microarray within 80 min, and the harvested daughter biopsies were expanded into viable hiPSC colonies suitable for downstream assays, such as polymerase chain reaction (PCR) or continued culture. Transmitted light microscopy offers an alternative, label-free modality for isolating hiPSCs, yet its low contrast and specificity for adherent cells remain a challenge for automation. This novel approach to label-free sensing and microcolony subsampling with the preservation of the mother colony holds the potential for hiPSC colony screening based on a wide range of properties including those measurable only by a cell destructive assay.

## I. INTRODUCTION

Induced pluripotent stem cells (iPSCs) hold the potential to revolutionize research in disease modeling, drug screening, tissue engineering, and personalized medicine by virtue of their ability to be readily differentiated into somatic cell types replicating the functions of primary cells. However, the production and maintenance of iPSCs from precursor cells such as erythroblasts or fibroblasts are complex, multistep processes that continue to require numerous manual steps. Further handling or manipulation of the cells requires similarly laborious quality control and sampling steps. Even in optimized culture conditions, iPSC cultures have a propensity for spontaneous differentiation, and differentiated cells must be identified for removal at the earliest stage to maintain high-quality cultures.^1^ Thus, accuracy in iPSC sensing is critical, not only to ensure safety in clinical applications but also to prevent failed cultures and reduce the costs of culture optimization.^2,3^ Microscopic observation provides a rich sensing modality for detecting iPSC-relevant phenotypes such as nuclear-to-cytoplasmic ratios, colony border definition, cellular compaction, apoptotic cells, and other morphologies.^4,5^ However, the expert manual microscopic observation of iPSCs remains the gold standard for iPSC sensing despite its limited throughput and precision.

Laboratory automation has the potential to address many limitations of current state-of-the-art iPSC maintenance and subculture, but current automated sensing strategies fall short of providing an effective, label-free, and robust method. To date, there is no all-in-one automated system that has demonstrated long-term label-free culture, handling, and sensing of viable human iPSCs (hiPSCs).^6^ Reported technologies for stem cell processing, such as tissue choppers, liquid handlers, laser microdissectors, suction aspirators, microfluidics, and microarrays, generally rely on the use of exogenous fluorescence labeling or specialized phase contrast microscopy-based methods for cell sensing.^7–15^ Exogenous labeling perturbs cells and requires either genetic engineering or cell- and experiment-dependent optimization of labeling compounds. Phase contrast approaches impose requirements on the imaging substrate, imaging matrix, and choice in optical hardware. A common challenge in traditional label-free transmitted light microscopy approaches lies in distinguishing transparent cells from transparent background surfaces and noncellular microscopic objects. The low image contrast and signal specificity are increased on microdevices with patterned microfeatures since the cells are forced to lie at varying focal planes and in regions with varying background transmissions. Thus, despite the development of technologies with exceptional cytometric and cell handling capabilities, the continued lack of robust, label-free microscopic cell sensing approaches precludes the application of these technologies for clinical, therapeutic, or disease modeling applications of hiPSCs.

Although various methods can perform highly accurate single cell segmentation from bright-field images via microscope defocusing, active contours, and level-set analysis, they come at a cost of high computational complexity.^16,17^ The extension of these methods for high-throughput detection on nonuniform backgrounds has not been reported. Few methods have been developed to meet the exceptionally high demands of computational efficiency and robustness required for automated and high-throughput bright-field mammalian cell detection.^18^ Buggenthin *et al.* achieved excellent throughput by detecting cells using maximally stable extremal regions, but the pipeline was not applicable to multicellular colonies.^19^ Chalfoun *et al.* developed an empirical gradient threshold approach with excellent accuracy for segmenting colonies of adherent cells; however, the method requires locally uniform backgrounds.^20^ Ultimately, current approaches for high-throughput cell sensing cannot effectively be applied to hiPSC colony sensing on patterned microdevices.

In this work, a label-free hiPSC detection method was developed which utilizes the image analysis of bright-field microscopy images to segment the colonies of adherent cells from nonuniform image backgrounds. The previously reported “quad” microraft array, which partitions each microarray element into four independently releasable subunits, was utilized to spatially isolate microcolonies expanding over the array.^21^ Specifically, adherent hiPSC colonies in culture on microraft arrays were accurately segmented from the images using a novel combination of standard deviation z-projections (SDPs) of images at multiple focal planes, robust background subtraction, and image texture analysis. After characterizing and validating the sensing method for hiPSCs and microrafts on quad microraft arrays, the image acquisition and analysis were automated and applied to the time-lapse monitoring of hiPSC cultures on the arrays. The sensing approaches were then implemented within a hardware and software platform for reacting to sensed microcolonies by performing autonomous, “smart” colony biopsy under computer control using microraft ejection. The main advances of the automated system involve the use of (1) the optical tracking of cell colonies as the cells spread across each of the microrafts within a quad colony site, (2) automated decision making to identify ideal microrafts to release for colony biopsy, and (3) the implementation of microscopy and image analysis which detect and optimize the physical ejection of microrafts from the array in real-time. Finally, we demonstrate the utility of the system for automated subculture by screening and biopsying over 200 hiPSC microcolonies.

## II. RESULTS AND DISCUSSION

### A. Overview of the sensing strategy and platform design

**FIG. 1.**
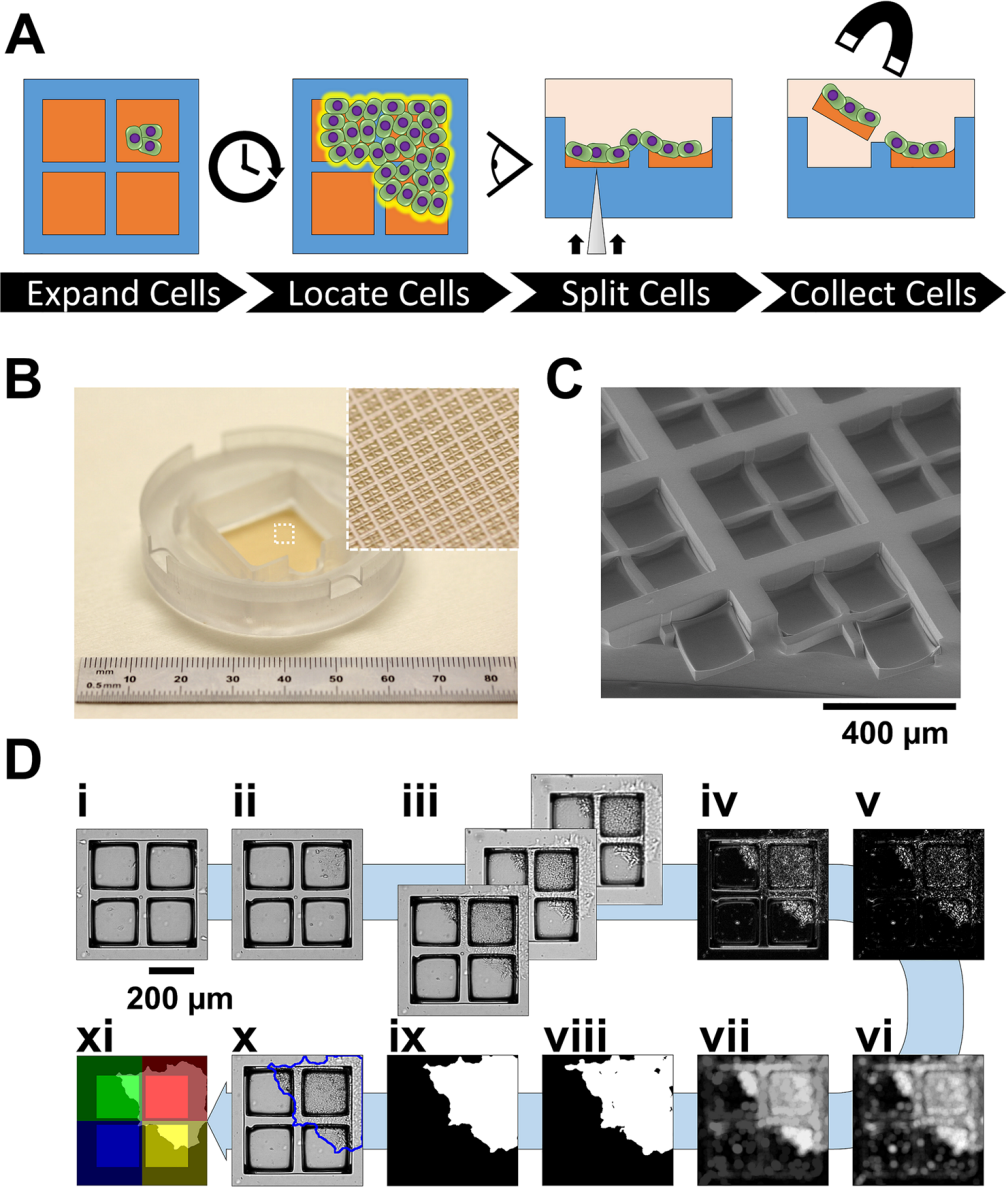
hiPSC sensing on microrafts. (a) Schematic overview of the platform for hiPSC sensing and isolation. (b) Photograph of a quad microraft array device; inset: macrozoom photograph of the microarray surface. (c) Environmental scanning electron microscope micrograph of the quad microraft array surface. (d) hiPSC sensing pipeline. Bright-field microscopy images of (i) cells seeded on one representative colony site; (ii) the expanding colony after 4 days of culture; and (iii) cells at varying microscope focal planes. Images were processed by (iv) background-subtracted standard deviation intensity z-projection (B-SDP), (v) top-hat filtering, (vi) entropy texture filtering, (vii) reconstructive opening, (viii) intensity thresholding, and (ix) morphological processing. Colony segmentations (x) were combined with the segmentations of the quad microrafts to spatially describe the colony's underlying microfeatures (xi). For visibility, image intensities were stretched to the brightest and dimmest 0.02% of pixels.

Several reports describe the use of slightly defocused bright-field imaging to enhance the contrast of adherent cells for straightforward cell detection (standard deviation z-projection or SDP).^22–24^ In particular, one method involved acquiring the bright-field microscopy images of cells from multiple focal planes, both focused and defocused, and calculating the SDP values along the focal axis to enhance the image contrast.^22^ The SDPs were expected to enhance the contrast since the intensity of pixels around and within cells varied with focus, while the intensity of the background remained relatively constant independent of focus. A platform based on motorized bright-field microscopy, microscale cell culture, and magnetic manipulation was chosen to develop, test, and apply SDPs for hiPSC sensing (Fig. 1). Motorized microscopy provided a framework for automation, while the microdevices permitted precise control over cell culture and manipulation.

Although vacuum suction or fluid flow is a viable approach to isolate and relocate cells, magnetic manipulation is ideal for handling sensitive cells such as hiPSCs because of the minimal direct forces. Various magnetic microcarriers have been reported such as magnetic micropallets,^25,26^ microtransporters,^27^ microplates,^28^ and microrafts.^29^ Microraft arrays are notable for their ease of manufacture and use, high biocompatibility, and exceptional compatibility with fluorescence microscopy and long-term cell culture. The arrays consist of micromolded cell carriers, typically composed of polystyrene with dispersed maghemite nanoparticles. The embedded superparamagnetic nanoparticles are ∼10 nm in diameter, below the diffraction limit of light, resulting in optically transparent microcarriers. To date, a wide range of cells have been isolated and assayed using the microraft arrays: primary intestinal stem cells,^30^ embryonic stem cell (ESC) and iPSC neurons,^31^ hippocampal cells,^32^ T cells,^33^ B lymphocytes,^34^ fibroblasts,^35,36^ colonic organoids,^37^ and various cancer cell lines including HeLa, H1299, AsPc-1-Luc, K-562s, and CFPAC-1 lines.^38–40^ The microrafts have been used for the isolation of cells for a wide variety of downstream assays with relevance to hiPSCs, such as polymerase chain reaction (PCR), ribonucleic acid (RNA) and whole genome sequencing, and immunocytochemistry.^31,34–36,38,41,42^

A new microraft design (quad microraft arrays) partitions magnetic microcarriers into clusters and offers the potential to section adhered colonies. To demonstrate this concept, quad microraft arrays were incorporated into a cell sensing and manipulation platform. The arrays were designed to match the culture area of one standard culture 6-well, with each colony site of sufficient size to accommodate 7 days of hiPSC growth. To achieve this, 1681 clusters of 2 × 2 microrafts (quads), each 200 *μ*m × 200 *μ*m in lateral size, were arrayed in poly(dimethylsiloxane) (PDMS) microwells (Fig. S1). To produce microrafts of sufficient durability for microneedle ejection, the microrafts were molded within 50-*μ*m-deep PDMS microwells to produce bowl-shaped microrafts averaging 39 *μ*m in thickness at the edges and 25 *μ*m thickness at the center. To maximize the colony site density, microrafts within the same quad were separated laterally by only 30 *μ*m of PDMS. The quads were separated by a 100-*μ*m-wide barrier of PDMS with a height 50 *μ*m above the microrafts. The culture surface of quad microraft arrays was multiplanar, making them a realistic and challenging environment for label-free hiPSC detection. The goal of the platform was to monitor hiPSCs in culture, locate expanded colonies, and isolate colony biopsies by releasing magnetic cell carriers and their attached cells for magnetic collection.

### B. Optimization and validation of microcolony sensing

Although SDPs have potential as tools for the label-free contrast enhancement of cells, the use of SDPs for adherent cell detection has not been systematically optimized nor has their use been evaluated for the detection of cells on uneven culture surface topographies. To evaluate the use of SDPs, an image analysis pipeline was developed to take advantage of the enhancement provided by SDP images to facilitate the digital segmentation of microcolonies from light microscopy data [Fig. 1(d)]. First, the focused and defocused bright-field microscopy images of hiPSCs in culture on quad microraft arrays were combined in SDPs.^22^ A defocusing amount of approximately 2× objective depths of focus (57 *μ*m) was chosen to create substantial visual variations between the images. The appearances of cells in the SDPs were characterized by bright, punctate spots within cell boundaries and bright halo outlines around the cell perimeter [Figs. 1(d-iv) and S2]. Next, a robust approach to background subtraction was taken to reduce the microarray image background that was present in raw SDPs. Since the visual appearance of the microraft array background remained constant over time during cell culture, its appearance was estimated from the bright-field images of the microraft arrays prior to cell seeding.^43^ The background images were then subtracted pixel-wise from images with cells at the same array position and focal plane. To facilitate this subtraction, all the raw images of the array were processed by flat-field and illumination corrections and subpixel translational and rotational registrations. SDPs from the background-subtracted images (“B-SDPs”) were top-hat filtered with linear structuring elements to eliminate long, straight edges belonging to microraft features. Entropy texture filtering was utilized to combine the cell-sized regions of speckled B-SDP signals into filled regions, marking patches of cells. Cellular debris was filtered by morphological size exclusion. Once processed, Otsu's intensity thresholding segmented the microcolonies. Since hiPSC colonies consisted of densely packed cells, small interior holes in the segmentation patches were considered artifacts and were morphologically filled. Finally, colony segmentations were morphologically thinned to compensate for the dilated appearance of cells in SDPs.

To validate the image enhancement pipeline, ground-truth hiPSC microcolony segmentations obtained from the fluorescence microscopy images of stained cells on quad microrafts were compared against the automated segmentation pipeline. The image pipeline increased the signal to noise ratio (SNR) of the processed images by 4.7 ± 0.5-fold over the raw bright-field images (N = 64 images). The segmentations had a pixel-wise true positive rate (sensitivity) of 88%, a true negative rate (specificity) of 98%, an accuracy of 98%, and a precision of 83% over 214 cell patches totaling 42.5 mm^2^ in area detected from a raster scan of 398 mm^2^ total microarray area. The Matthews correlation coefficient (MCC), a balanced metric of classification accuracy, was 0.84, indicating a strong correlation between the automated and ground truth colony segmentations.^44^ It should be noted that automatically detecting hiPSC colonies in images with locally uneven backgrounds is a novel application, without comparable methods in other literature reports. Nevertheless, the SDP pipeline's metrics indicated a good baseline segmentation performance, especially since reported methods for cell segmentation without uneven image backgrounds demonstrate accuracies, specificities, and sensitivities ranging from 85% to 95%.^19^

Automated bright-field cell detection can be highly useful for many bioanalytical technologies. To explore the flexibility of our pipeline, the performance of automated segmentation was evaluated using the images of adhered colonies on quad microraft arrays of a second cell type, HEK-293T. HEK-293T cells are known for their ease of transfection, making them common targets for genetic engineering and desirable cell types for microarray-based sensing and screening. Like hiPSCs, they adopt an epithelial morphology in adherent cell culture. The HEK-293T cells were fixed on a quad microraft and imaged with objectives including 4×, 10×, and 20× with defocusing amounts at 2 *μ*m intervals from 2 and 100 *μ*m above and below the focal plane. Again, fluorescence microscopy images served as the ground truth for colony segmentation. In all cases, the sensitivity, specificity, and MCC of the segmentations exceeded 84% regardless of objective choice as long as the chosen defocusing was more than 8 *μ*m. This result suggests that the combined enhancements of background subtraction, SDP, and subsequent calibrated image filters were sufficient to grant the analysis pipeline relative insensitivity to input image appearance and quality as the cell type, objective, and defocusing amount were varied.

### C. Evaluation of microraft detection and tracking

To complement the cell sensing method, a microraft sensing method was developed to enable the unique identification of adhered colonies in terms of their underlying microrafts. Microraft arrays are ideal culture surfaces for cell tracking due to their uniform grids of elements, enabling the spatial indexing of attached cells [Figs. 1(b) and 1(c)]. A recent, automated approach demonstrated that, by combining automated microscopy and digital image analysis, precise microraft positions and identities could be determined and tracked over time.^33,39^ This approach was adapted for quad microraft arrays (Fig. S3). Specifically, quad microrafts were identified from bright-field microscopy images by processing them with flat-field correction, intensity threshold binarization, and morphological filtering. Then, located microrafts were virtually indexed by their row and column positions relative to the overall microarray grid. Using this approach, the microrafts' centroids, image data, and unique identifiers were linked.

To evaluate the effectiveness and robustness of the microraft sensing and tracking approach for the quad microrafts in the presence of cells, Matrigel-coated microraft arrays were seeded with adherent hiPSCs and imaged daily for 5 days of culture. The arrays were raster-scanned with a motorized microscope automated using MATLAB scripts.^45^ The microraft segmentation proceeded robustly when cells were present in the images, with >92% of colony sites detected for all timepoints. The segmented colony sites measured a side length of 431 ± 8 *μ*m compared to the designed 430 *μ*m. All colony sites not occluded by the media chamber were “tracked”—assigned a unique index to allow their positions to become matched across all timepoints. The tracking algorithm was designed to interpolate any missing positions and/or indices of microrafts as needed. The algorithm maintained tracking of 94% of the colony sites not occluded by the media chamber when it was artificially forced to interpolate up to 67% randomly deleted microraft positions and indices. The results indicate that automated microscopy imaging and image analysis were efficient and robust tools to sense and track quad microrafts.

### D. Application to hiPSC culture monitoring on quad microraft arrays

**FIG. 2.**
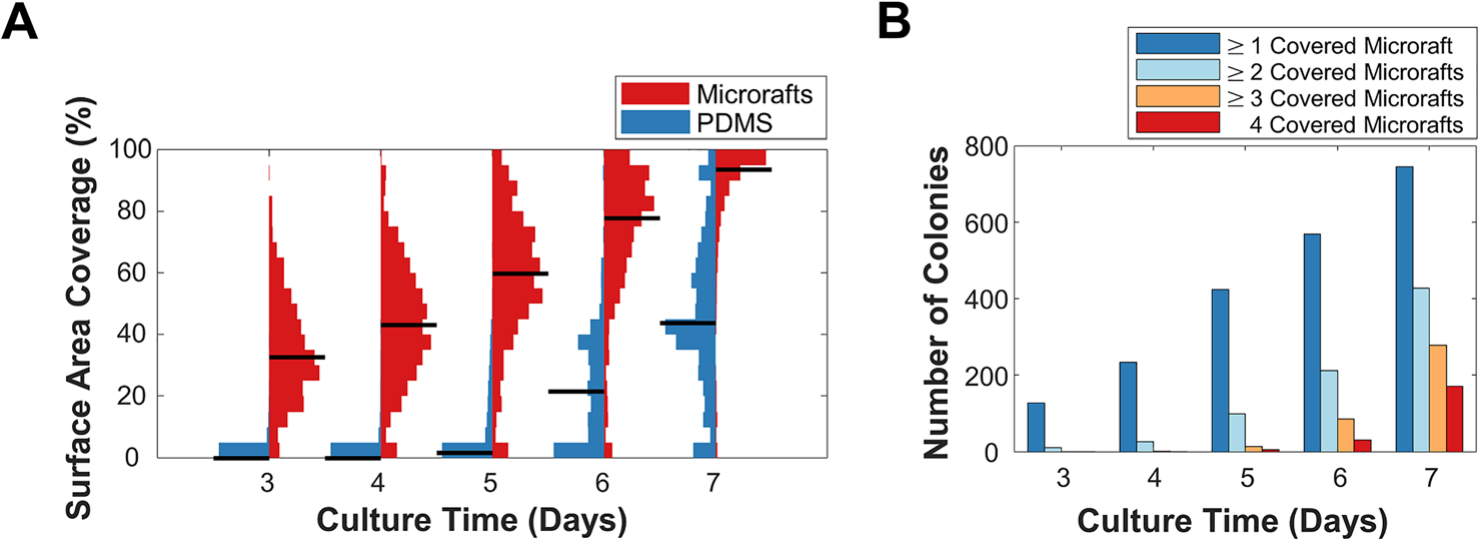
Cytometry of hiPSC microcolony confluency on microraft arrays using label-free bright-field cell sensing. (a) Violin plots showing the progression of microcolony coverage over microraft and PDMS surfaces of quad microraft arrays. Black lines denote the median of each distribution. The total width of each histogram was normalized to the histogram's maximum width bin. (b) Classification of hiPSC colonies by the number of microrafts covered by each colony (coverage defined as ≥50% surface area) over time.

The methods for sensing hiPSCs and microrafts were combined to quantitatively track the size of hiPSC colonies in culture on a microarray. A quad microraft array seeded with hiPSC clusters (on average four cells per cluster) was cultured for 7 days. The microarray was imaged prior to seeding and daily thereafter. The images were processed by the analysis pipeline to first segment the hiPSC colonies and microrafts and then to extract temporal-spatial data on each detected colony's location and size. By day 3 of culture, the detectable hiPSCs were located primarily on the microrafts rather than PDMS [Fig. 2(a)]. Over time, cells expanded across their underlying microraft and over the minimal PDMS barrier to adjacent microrafts within their quad [Fig. 2(b)]. As further validation of the pipeline, the 1807 colonies at day 5 of culture were hand-segmented. Compared to manual segmentations, the automated segmentations had a pixel-wise segmentation sensitivity, specificity, and MCC of 80%, 99%, and 0.83. The difference in the measurements of the colony area, measured as the median absolute difference in the areas of the 1110 colonies of radius ≥25 *μ*m, was 10%. The final classification of colonies as having spread over 1, 2, 3, or 4 microrafts was 84% accurate. Overall, thousands of microrafts were screened for cells, and the sizes of hundreds of microcolonies were tracked over seven timepoints, demonstrating the utility of quad microraft arrays for parallel hiPSC microcolony culture and the scalability of the bright-field sensing method.

### E. Quad microraft release with real-time optical feedback

**FIG. 3.**
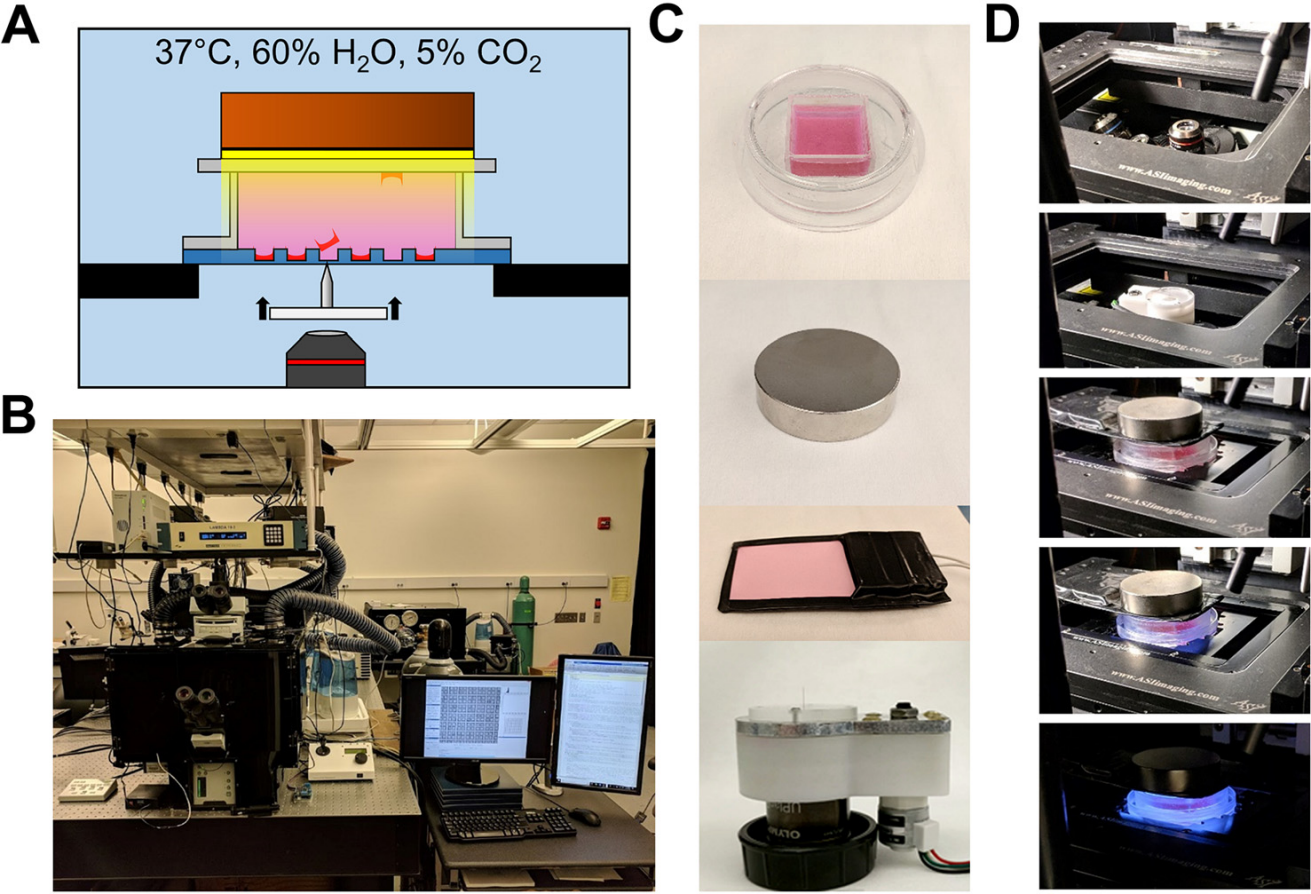
Automated microraft release system. (a) Schematic of the automated microraft release approach. Superparamagnetic microrafts (red) are positioned over a microneedle using a motorized X-Y stage, after which the microneedle is actuated to dislodge the microrafts from the PDMS microarray substrate (blue). Dislodged microrafts are pulled upward through liquid media (pink) by a magnet (brown) placed above the microdevice. An EL panel (yellow) illuminates the microarray, enabling an inverted microscope to collect the visual feedback about the location of microrafts throughout the process. (b) Motorized IX81 microscope, incubator, and PC setup. (c) From top to bottom: quad microraft array, neodymium disk magnet, EL light panel, and motorized microneedle device. (d) Assembly of hardware on the motorized microscope, in sequence from top to bottom.

The B-SDP method generated high-throughput microcolony cytometry and tracking data, which if paired with high-throughput quad microraft isolation would enable hiPSC colony subsampling. A platform for the automated isolation of nonadherent cells on microrafts has been previously reported which was capable of isolating microrafts at a rate of approximately 100 per hour.^39^ In order to expand the abilities of microraft isolation technologies, a robust, image-guided automated platform designed specifically for the novel and challenging task of adherent colony sampling was developed (Fig. 3). The core of the system was a quad microraft array culture microdevice arranged on the stage of an incubated motorized microscope stage. To maximize the rate of magnetic microraft collection, ejected microrafts with cells were captured into a Petri dish situated directly above the array using the attraction of a disk magnet. A thin electroluminescent (EL) sheet was incorporated between the magnet and the microarray to illuminate the microarray without displacing the magnet. Finally, a motorized microneedle above the microscope objective enabled simultaneous microscopy and microraft dislodgement.

**FIG. 4.**
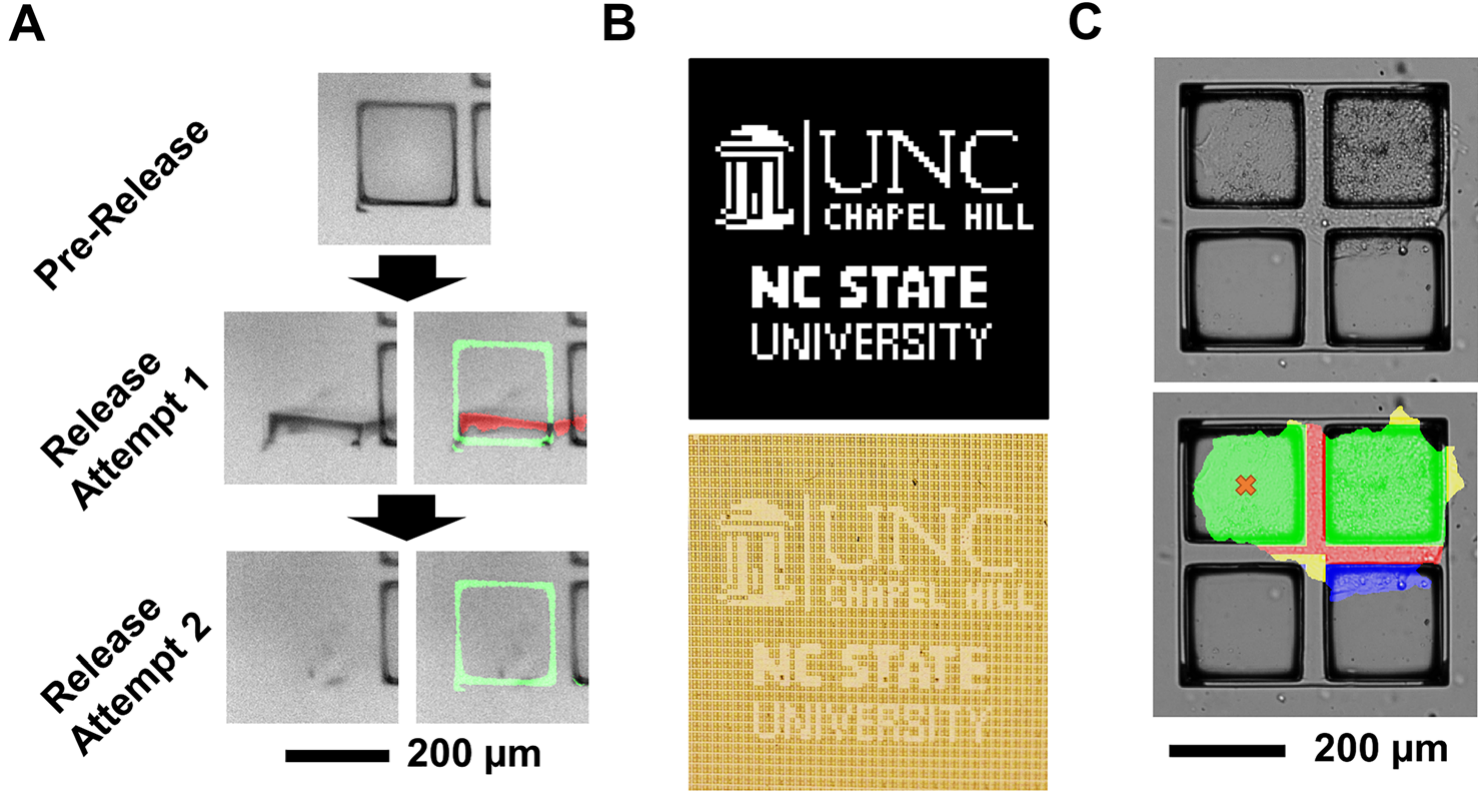
Automated targeting and release of quad microrafts. (a) EL-illuminated bright-field microscopy images of a microraft during the automated release process. Green overlay: detection of the dislodged microraft material. Red overlay: detection of the remaining microraft material. (b) University logo pattern (top) replicated on quad microraft arrays (bottom) via high-throughput, automated microraft release. (c) Microraft selection. The bright-field microscopy images of hiPSCs on a quad colony site (top) were processed by the colony and microraft segmentation pipelines to locate cells and microfeatures (bottom). Green and blue: colony sections with ≥ and < 50% raft coverage, respectively. Red and yellow: ≥ and < 30% PDMS border coverage. The orange cross marks an optimal target microraft for splitting the colony, automatically chosen for its minimal PDMS coverage.

An automation process was designed to maximize the microraft sampling rate and efficiency (Fig. S4). Once the magnetized and illuminated capture dish was seated above the microarray, the motorized microscope scanned the microraft array using the transmission of the EL light source to relocate all microrafts in the array. Then, given a list of target microraft indices on an array, the software optimized the order of the microraft targets according to a Nearest-Neighbor algorithm to minimize the total travel distance required by the hardware to traverse and release all of the targeted microrafts from the array (Fig. S5). Next, the motorized stage moved the microarray such that its current targeted microraft was centered over the microneedle. An image was acquired using the EL light source at this location to recalculate the centroid of the targeted microraft by intensity thresholding. The microneedle was then actuated to pierce the PDMS array substrate and dislodge the target microraft from its microwells. The microneedle was retracted, and the microscope reimaged the targeted location. The image was again processed by intensity thresholding, and the extent of microraft dislodgment was measured from the ratio of the super-threshold pixel area to the total microwell pixel area [Fig. 4(a)]. If the dislodgment extent was less than 80%, the position of the presumably undislodged microraft was measured as the area-weighted centroid of supra-threshold pixels. The microraft release process was then repeated by aiming at this centroid and continuing to iterate until concluding for each target when the dislodgement extent exceeded 80% or the number of attempts exceeded eight.

To evaluate the baseline precision and throughput of the automated release, 683 microrafts from an array without adhered cells were targeted for release in the pattern of university logos [Fig. 4(b)]. The microraft release was >99% efficient, with no off-target microrafts released. The system performed 1.89 ± 0.04 ejection attempts per microraft, with 93.6% of the microrafts dislodged after two attempts. The release required 112 min, including software startup times (Table I). The system measured and compensated for 2.5 ± 3.0 *μ*m of displacement error in the stored microraft locations. The image analysis of PDMS membranes punctured by the system indicates that there was a negligible variation in the microneedle puncture location across the microarray: 13.9 ± 9.5 *μ*m displacement error (Fig. S6). Overall, the system metrics indicated that the automated platform was capable of rapid, robust, and high-throughput release of quad microrafts.

**TABLE I.** Temporal breakdown of automated microraft release.

Subprocess	Total time (min)	Iterations
Microarray shape modeling	2.6	1
Locating microrafts	1.7	1
Identifying microrafts	1.0	1
Autofocusing	6.9	28
Needle positioning	5.3	683
Needle actuation	37.4	1185
Image guidance	56.6	1185

### F. hiPSC microcolony splitting on quad microrafts

**FIG. 5.**
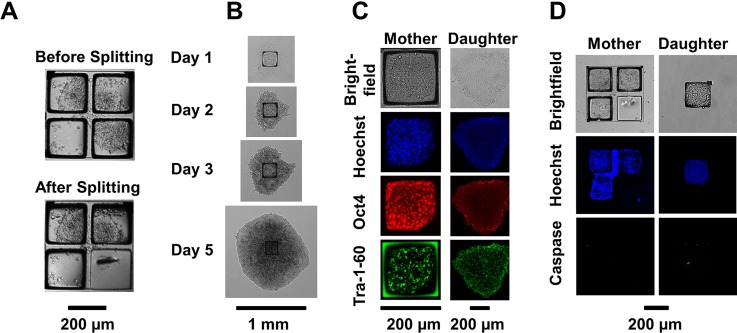
hiPSC colony splitting and viability on quad microraft arrays. (a) Bright-field microscopy images of a microcolony before and after being split via the removal of the lower right microraft by microneedle ejection. (b) Timelapse images of expanding hiPSCs split from microraft arrays. (c) Immunofluorescence microscopy images of pluripotency biomarkers expressed by mother hiPSCs in culture on a microraft and daughter cells after biopsy, expansion, and one passage in a well plate. (d) Caspase-3/7 staining of split hiPSC colonies (green), with mother cells on the microarray and daughter cells on a microraft magnetically isolated into a well plate.

We combined microraft-based mechanical colony splitting with nonenzymatic cell dissociation to develop a method for hiPSC subcultivation on microraft arrays. Intercellular connections spanning the PDMS walls were weakened by preincubation of the arrays in ethylenediaminetetraacetic acid (EDTA), and then, the microrafts bearing cells were released from the array using a motorized microneedle. In contrast to previous methods,^46^ our hybrid approach utilized a brief EDTA incubation to encourage the subsequent mechanical splitting by the microraft ejection. Notably, microcolonies split along the microraft edges even if their cells extended off the microraft [Fig. 5(a)]. In a preliminary test, 63% (14/22) of split events completely separated the targeted microraft from the array, thus effectively biopsying the colony. Cells of split colonies remained adherent to their microrafts, which is consistent with the known impact of brief EDTA treatments. These results suggested the feasibility of subsampling of cell microcolonies cultured on the quad arrays via the selective ejection of microrafts.

To investigate the impact of microraft-based colony splitting on the hiPSCs, the capability for self-renewal, the retention of pluripotent stem cell biomarkers, and the occurrence of apoptosis were examined before and after the nonenzymatic mechanical colony splitting process. After microcolony biopsy via microraft release, colony fragments on microrafts adhered to fresh Matrigel-coated polystyrene dishes and rapidly proliferated to form dense, radially expanding hiPSC colonies [Fig. 5(b)]. The doubling time of the total colony areas of postmicroraft-biopsy hiPSCs and untreated hiPSCs was calculated from linear fits of 72 hours of log-linear growth in 6-well plates to be 17 h [13 h, 23 h] ([95% confidence interval]) and 18 h [15 h, 23 h], respectively (Fig. S7). hiPSCs cultured on the Matrigel-coated quad microraft arrays lacked morphological signs of differentiation and consistently expressed Oct4 and Tra-1–60 pluripotency markers [Fig. 5(c)]. These biomarkers were quantified because their absence would indicate a loss of pluripotency. On the microrafts, 98% of cells were Oct4^+^ and 94% were Tra-1‐60^+^ (N ≥ 82 000 cells), compared to cells on standard culture ware with 94 ± 3% Oct4^+^ cells and 91 ± 3% of Tra-1‐60^+^ cells (N = 3 biological replicates of 11 000 cells). The pluripotency markers and stem-like morphology were retained in hiPSCs split by microrafts and in split cells that were expanded and passaged. In the expanded colonies, 97 ± 2% of cells were Oct4^+^ and 91 ± 8% were Tra-1–60^+^ (N = 3 biological replicates of 50 000 cells). The expression rate of Tra-1‐60 in cells passaged using microrafts was equivalent or greater than literature reports from cells passaged by other automated technologies, namely, 80%–91%, 84%, and 85%–95% for liquid handling, laser ablation, and capillary selection, respectively.^9–11^ Since hiPSCs are prone to anoikis, or cell-dissociation induced apoptosis, Caspase-3/7 was used as a marker of early apoptosis in the hiPSCs [Fig. 5(d)]. A baseline of 96.4% of cells on the microarray was Caspase-3/7^−^ (N ≥ 83 000 cells), compared to cells on standard culture ware with 99.9% ± 0.04% (N = 3 biological replicates of 23 000 cells) of Caspase-3/7^−^ cells. Similarly, 94.9% of split mother cells were Caspase-3/7^−^ (measured over 2000 cells) and 97.0% of daughter cells (measured over 600 cells) were Caspase-3/7^−^. Taken together, these results are evidence for the retention of viability and proliferation capabilities of hiPSCs throughout microraft-based microcolony culture and colony splitting.

### G. Application to automated hiPSC subculture using quad microrafts

A common requirement for many bioassays is the ability to split cells into two or more fractions: at least one to undergo a cell-destructive assay or a quality control test and the other to retain for continued culture or biobanking. As a proof-of-concept, the automated cell sensing and hiPSC colony splitting system was applied to split daughter cells from microarrays for expansion culture while retaining intact mother cells on the array. Specifically, a Matrigel-coated quad microraft array was seeded with hiPSCs as multicellular clusters, cultured for 5 days, and imaged daily at focal planes optimized for the SDP. On the final day of culture, every quad colony site was considered for release as long as it contained >1 microraft with more than 50% detected cell coverage—a target criterion that was selected to maximize the biopsy survival and outgrowth rate. For quads with multiple possible biopsy targets, the microraft with the least cellular outgrowth onto the adjacent PDMS surface was targeted, with the assumption that these microrafts would be the most readily released [Fig. 4(c)]. Additional release criteria were utilized to prevent excessive release attempts in situations where microrafts were dislodged from the array, yet they remained loosely or temporarily connected to the array by cell-cell junctions. Specifically, the release attempts were halted when the centroid of the dislodged microraft was >70 *μ*m away from its initial location or when a comparison of the pre- and postrelease images identified more than 1/3 of the targeted microraft outside of its microwell. The automated system dislodged one cell-bearing (>50% microraft surface coverage or confluency) microraft from every quad sensed to contain a colony (≥2 microrafts with >50% confluency).

**FIG. 6.**
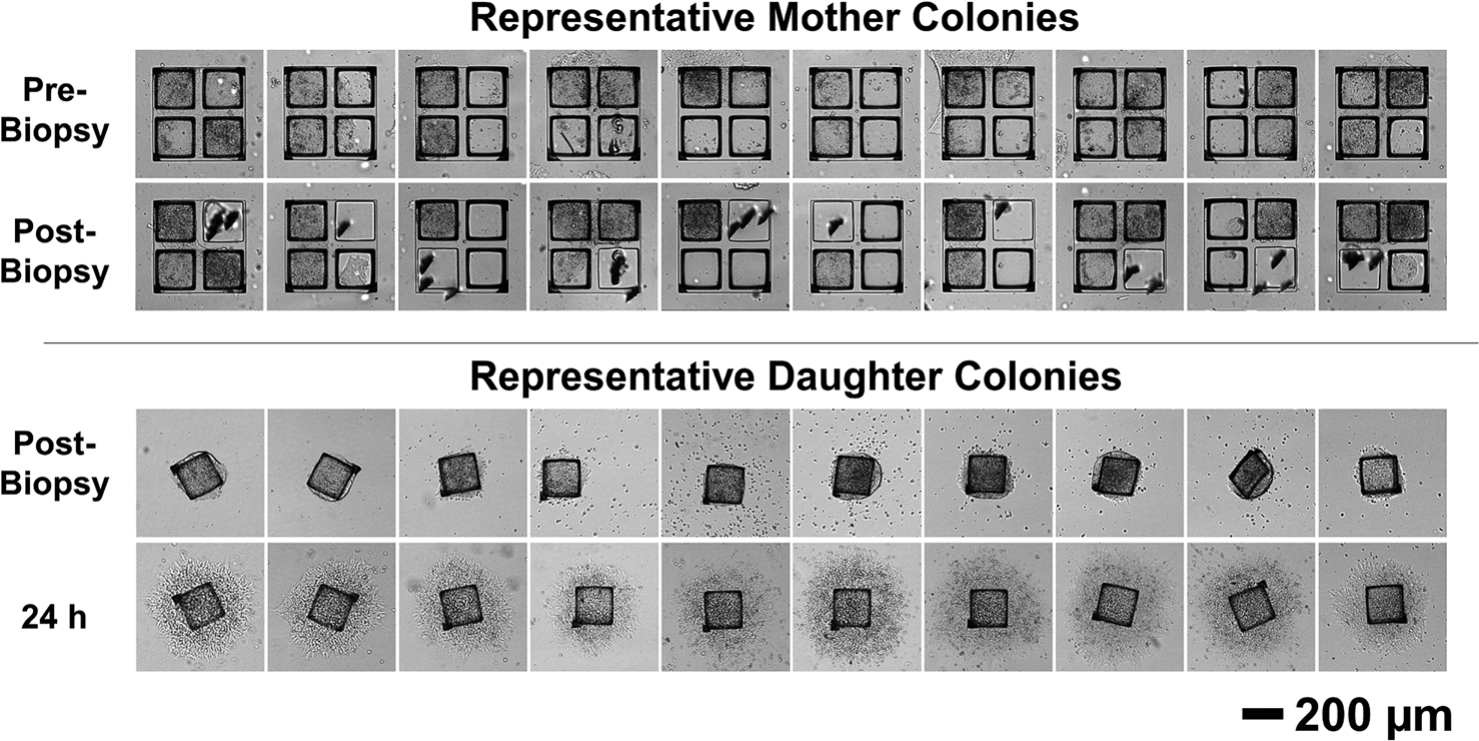
Automated hiPSC biopsy using quad microraft arrays. The bright-field microscopy images of representative mother and daughter colonies before and after microcolony splitting.

A total of 231 microrafts were identified as release targets, with 140, 36, and 55 of the targets located within colonies with cells sensed over two, three, and four microrafts, respectively. Later manual inspection confirmed that 99.5% of the automatically sensed targets had adhered cell loads that exceeded the 50% confluency criterion. The system successfully dislodged the target microrafts over a period of 77 min with, on average, 4.2 ± 1.6 dislodgement attempts made per target. The assessment of the microarray confirmed that 97.5% or 225 of the targeted microrafts were released from the microwells. Ultimately, 73.6% or 170 of microraft targets were effectively removed from the array while also leaving behind microrafts containing the corresponding mother cells at the original quad colony site (Fig. 6). The remainder of targeted microrafts (26.4%, 61 microrafts) were also removed from the array but dislodged additional cells from the mother colony in the process, leaving behind insufficient cells for the survival of the mother fragment. A fraction of the collected material, consisting of 34 microrafts with cells, was transferred to a 6-well plate where the low density of microrafts facilitated the quantitative evaluation of each colony over time using time-lapse microscopy imaging. All 100% (N = 34) of the monitored daughter microcolonies rapidly spread beyond their 200 × 200 *μ*m microraft carriers to form large hiPSC colonies which, after 4 days of culture, were 1080 ± 230 *μ*m in diameter (Fig. S8). Overall, these results indicate the capability to rapidly and automatically subculture hiPSCs with high cell viability using quad microraft arrays, bright-field cell sensing, and hardware automation.

The microraft array passaging of iPSCs compares favorably to other automated technologies. Terstegge *et al.* have reported laser microdissection methods capable of precise isolation of selected cells, which biopsied iPSCs at a rate of 10 s per sample, comparable to the microraft method.^10^ However, the laser isolation damaged iPSCs in the process, and the reported 36% colony survival rate indicates the limited applicability of the method for high-throughput applications such as iPSC passaging. Haupt *et al.* have reported an improved, yet still suboptimal, post-sort iPSC colony survival rate of 76% with a vacuum-suction-based selective cell picker which was also reported to operate at a slower rate of 50 s per sample.^11,47^ In contrast, liquid handling platforms for iPSC passaging can simultaneously maintain several hundred iPSC lines with exceptional cell viability, but they are unable to selectively passage cells because they perform bulk chemical or enzymatic passaging.^8,9^ In comparison to these three technologies, microraft-based passaging is distinguished by its use of image guidance to gently and selectively passage miniaturized, addressable hiPSC colonies. The relevance of image-guidance for selective cell passaging continues to increase with the development of new methods for image analysis, such as machine-learning approaches for detecting spontaneous differentiation from the bright-field images of stem cells.^48–50^ Microraft array passaging benefits from the minimal reagent consumption from the microcolony culture format. The method scales with both the cell culture area and the rate of colony splitting. For example, the number of hiPSC lines can be increased by housing 144 of the 24 × 24 mm arrays, each containing one cell line, per microscope stage. By staggering the cultures over 48 h, the 144 clones could be passaged using a 3:1 subcultivation ratio at a conservatively estimated rate of 20 min per array using currently available hardware. Previous research has demonstrated the use of magnetic wands to relocate live-cell-laden microrafts into other culture vessels such as PCR tubes or multiwell plates.^33,38,39^ This capability opens the possibility to use microrafts to reformat passaged hiPSCs/microrafts into other types of secondary vessels for further culture, assays, or integration into other automated instruments.

## III. CONCLUSION

The B-SDP image analysis pipeline represents a straightforward method for detecting adherent cells on surfaces with microfeatures. This sensing strategy is well situated for detecting and monitoring sensitive cell types such as primary or stem cells since the lack of labeling simplifies experimental workflows and minimally perturbs cells. The B-SDP method distinguishes cells from an arbitrary background based upon their respective changes in appearance at different focal planes, without the need for training datasets. The pipeline is promising for cell detection in the presence of a variety of backgrounds, such as those observed in microfluidic devices, microarrays, micropatterned surfaces, and microengineered culture scaffolds. Overall, this approach is notable for its sensitivity and specificity, relatively simple computational load, low calibration requirements, and robustness. In this work, cell sensing readouts were limited to microcolony size, location, and confluency, but these fundamental metrics could set the framework for the extraction of more complex measurements. For instance, the B-SDP approach could be used to segment and pre-label the microcolonies to the facilitate subsequent machine learning classification analyses of hiPSC density or differentiation.

Microraft arrays are versatile tools for single cell cytometry and cellular isolation. Here, the capabilities of the microarrays were shown to extend to colony-based assays of adherent cells. The additions of real-time visual feedback for microraft speed and reliability and algorithms for smart microraft targeting set a new standard for microraft array automation. The ability to automate microcolony splitting without labeling cells has the potential to facilitate a wide range of automated bioassays especially those in which a cell-destructive method is required for cell assay. The biopsy of living colonies, with handoff of the colony fragment(s) for assays such as PCR, RNA-seq, or immunostaining, enables the living mother microcolony to remain on the array for later harvesting.

## IV. METHODS

### A. Reagents

A poly(dimethylsiloxane) (PDMS) Sylgard 184 silicone elastomer kit was purchased from Dow Silicones Corporation (Midland, MI). Poly(acrylic acid) (PAA) (MW 30,000, 30% in H_2_O) was obtained from Polysciences, Inc. (Warrington, PA). ReLeSR and mTeSR-1 were procured from STEMCELL Technologies (Vancouver, BC, Canada). Ethylenediaminetetraacetic acid (EDTA), Dulbecco's Modified Eagle Medium (DMEM), and human embryonic stem cell-qualified Matrigel were acquired from Corning (Corning, NY). SC102-A hiPSCs were purchased from System Biosciences (Palo Alto, CA). HCL4517 HEK-293T cells were procured from GE Healthcare (Little Chalfont, England). Phosphate buffered saline (PBS), bovine serum albumin (BSA), penicillin/streptomycin, Triton X-100, Hoechst 33342, CellEvent Caspase-3/7 Green, and Alexa Fluor 488 secondary antibody (A10667) were obtained from Thermo Fisher Scientific (Waltham, MA). Fetal bovine serum (FBS) was procured from Atlanta Biologicals (Flowery Branch, GA). Gamma-butyrolactone (GBL), paraformaldehyde, glycine, and Tween-20 were procured from Sigma-Aldrich (St. Louis, MO). Sodium azide (NaN_3_) was acquired from VWR (Radnor, PA). Primary antibodies for Oct4 (ab198570) and Tra-1-60 (ab16288) were purchased from Abcam (Cambridge, MA). Alexa Fluor 647 (711-605-152) secondary antibody was obtained from Jackson ImmunoResearch Inc. (West Grove, PA). Y-27632 dihydrochloride (ROCK inhibitor; ROCKi) was procured from ApexBio.

### B. Microraft ejection device

The motorized microneedle device was designed as previously reported and utilized a stainless steel microneedle (8 mm total length, 100-*μ*m sheath diameter, 500-*μ*m taper length, and approximately 5-*μ*m tip diameter) seated and epoxy-sealed into an acrylic window oriented above an objective.^39^ The window was incorporated onto a cantilever that was actuated up and down by means of a stepper motor (Ametek, Berwyn, PA). The microneedle device was controlled using an Arduino Uno with a motorshield attachment (Adafruit Industries, New York, NY) and was automated by MATLAB scripts using the MATLAB Support Package for Arduino Hardware to enable communication with the Arduino by serial commands over Universal Serial Bus (USB). For automated microraft release experiments, a 2 in. diameter, ½ in. thick N52 disk magnet (K&J Magnetics, Pipersville, PA) was used to collect all microrafts with biopsied colonies. An electroluminescent (EL) sheet (Electro Luminescence Inc., Aromas, CA) was used to illuminate the array during biopsy.

### C. Microscopy

Imaging was performed using a motorized IX81 microscope (Olympus Corporation, Tokyo, Japan) equipped with a MS-2000 motorized stage (ASI, Eugene, OR) and a Flash 4.0 V2 camera (Hamamatsu, Shizuoka, Japan). The microscope was situated within a Plexiglas incubator that maintained a temperature of 37 °C, with 60% humidity and 5% CO_2_. The objectives used were Olympus UPLFLN 4× (NA 0.13), 10× (NA 0.3), and 20× (NA 0.45). The following fluorescence filter cubes were utilized: Chroma ET-DAPI 49000, Semrock TxRed-4040B, Chroma ET-Cy5 49006, and Semrock FITC-3540B. All microscopy was automated using a graphical user interface (GUI) written in MATLAB (2018A, Math Works, Natick, MA) that controlled the automated components using Micro-Manager's Java API.^51^ All focusing was performed using a published software autofocus routine that utilized a modified Laplacian focus measure.^45,52^ Raster-scan microscopy imaging was performed with an image overlap equivalent to 600 *μ*m (equivalent to 1.1 quads) and at the focal plane estimated from the models of the curvature of microraft arrays generated as reported previously.^45^ During microraft releases, new focal data were acquired every 15 min to update the models and compensate for potential sample drift along the focal axis.

### D. Image analysis

Raw bright-field images were flat-field corrected and normalized to the mean intensity of the first image of each time-series. Flat-fields were approximated by mean-filtering raw microscopy images in 256 × 256 (415 × 415 *μ*m) blocks and then Gaussian smoothing with a 100 pixel (163 *μ*m) kernel standard deviation.^53^ Otsu's automatic intensity thresholding was used to segment microrafts from the background of corrected bright-field images.^54^ The thresholded images of microrafts were morphologically processed to produce segmentations of microrafts and quads. Specifically, interior holes in binary objects were filled, objects at the image border were removed, adjacent quad subunits were merged using morphological closing, and size exclusion was applied to minimize false positive detection. Measured quad centroids were assigned a digital identifier in the form of row and column numbers relative to the microarray grid. Starting from the real-world x-y coordinates of detected microrafts, any identified centroid duplicates were consolidated by averaging all centroids closer than 430 *μ*m (equivalent to 0.8 quad elements). The centroids of undetected microrafts were estimated by interpolation along rows and columns of the uniform microarray grid. A piecewise linear interpolation in 8 mm segments (equivalent to 15 quad elements) was chosen to compensate for potential array distortion. Finally, starting from the top left microraft array, row and column indices were assigned to each microraft.

Cell colony segmentations were generated by using a MATLAB image-processing pipeline. First, bright-field images were acquired at three focal planes of a sample both prior to cell seeding (background) and at the culture time of interest (signal). The raw images were processed by flat-field and illumination corrections as described above. Next, the background images were registered to the images with cell signals using the single-step discrete Fourier transform algorithm with a cross correlation error metric, an algorithm capable of subpixel accuracy, and fast computation time.^55^ The translational registration was iterated over –0.5° to 0.5° when determining the angular registration. After registration, the SDP was calculated by computing the pixelwise standard deviation of intensity along the Z-stack axis as described previously.^22^ The resulting background-subtracted SDP (B-SDP) images were top-hat filtered with a rectangular structuring element of 2 × 33 *μ*m (1/6 the length of a microraft) oriented at 0° and 90°.^56^ A local entropy texture filter with a 14-*μ*m radius disk element was applied to associate punctate B-SDP signals from adjacent cells. Morphological opening with reconstruction and a 40-*μ*m radius disk element was performed to exclude cell debris and to smooth the image. Segmentation was performed using Otsu's automated intensity thresholding. Interior holes exceeding 14 *μ*m in radius were filled morphologically, and the segmentations were thinned by 8 *μ*m and processed by a majority filter.^57^ The sizes of the morphological elements were chosen to segment the colonies of cells into contiguous objects.

The biomarker fluorescence intensity was measured and normalized by measuring the area of the biomarker fluorescence (above a threshold intensity) and dividing by the total area of the nuclei as measured by the area of Hoechst fluorescence (above a threshold intensity).

### E. hiPSC colony splitting on microraft arrays

Quad microraft arrays with adhered hiPSCs were incubated at 37 °C with 500 *μ*M EDTA for 2–5 min, depending on the amount of cells, to weaken cellular connections prior to microraft-based cell biopsies. Biopsies were performed by the microneedle ejection of the underlying microrafts using a microneedle translation speed of 2 mm/s. Subsequent gentle flushing with media (3× flush of 2 ml) or cell scraping (Fisher #08–100-241) was performed to detach weakly adherent overgrown cells in the microarray barrier regions. For automated hiPSC biopsy experiments, the dislodged cell-bearing microrafts were transferred to Matrigel-coated 6-well plates for expansion culture by gently flushing the microraft array with media followed by liquid transfer into the wells with a 5 ml serological pipette.

### F. Statistics

Unless otherwise noted, measurements are reported as the mean ± sample standard deviation. Confidence intervals on linear regression parameters were evaluated at a significance level α of 5%.

### G. Ethics

No ethics approval was required for this text.

## SUPPLEMENTARY MATERIAL

See the supplementary material for the descriptions of the established methods and for supplementary figures.
